# Outbreak of cholera in the Southwest region of Cameroon, 2021-22: an epidemiological investigation

**DOI:** 10.1186/s12889-024-21126-z

**Published:** 2024-12-25

**Authors:** Eugene Bangwen, Jane-Francis Tatah Kihla Akoachere, Daniel Mabongo, Adeline Bime, Elise De Vos, Marie Meudec, Wilfred Ngwa, Jerome Fru-Cho, Linda Esso, Marianne van der Sande, Brecht Ingelbeen, Soledad Colombe, Laurens Liesenborghs

**Affiliations:** 1 Institute of Tropical Medicine Antwerp, Nationalestraat 155, 2000, Antwerp, Belgium; 2 Department of Microbiology, Immunology and Transplantation, KU Leuven, Leuven, Belgium; 3 Department of Microbiology and Parasitology, Faculty of Science, University of Buea, Buea, Cameroon; 4 Southwest Regional Delegation of Health, Buea, Cameroon; 5 epiGuider Company LTD, Yaoundé, Cameroon; 6 Department for the Control of Diseases, Epidemics and Pandemics, Ministry of Public Health, Yaoundé, Cameroon; 7 Faculty of Medicine and Biomedical Sciences, University of Yaoundé I, Yaoundé, Cameroon; 8 Julius Centre, Global Public Health and Bioethics, University Medical Centre Utrecht, Utrecht University, Utrecht, Netherlands

**Keywords:** Cholera outbreak, Cross-border spread, Risk factors, Conflict, Humanitarian crisis

## Abstract

**Background:**

In October 2021, a large outbreak of cholera was declared in Cameroon, disproportionately affecting the Southwest region, one of 10 administrative regions in the country. In this region, the cases were concentrated in three major cities where a humanitarian crisis had concomitantly led to an influx of internally displaced persons. Meanwhile, across the border, Nigeria was facing an unprecedented cholera outbreak. In this paper, we describe the spread of cholera in the region and analyse associated factors.

**Methods:**

We analysed surveillance data collected in the form of a line list between October 2021 and July 2022. In a case-control study, we assessed factors associated with cholera, with specific interest in the association between overcrowding (defined by the number of household members) and cholera.

**Results:**

Between October 15, 2021 and July 21, 2022, 6,023 cases (median age 27 years, IQR 18–40, 54% male) and 93 deaths (case fatality 1.54%) were recorded in the region. In total 5,344 (89%) cases were reported from 6 mainland health districts (attack rate 0.47%), 679 (11%) from 4 maritime health districts (attack rate 0.32%). More than 80% of cases were recorded in 3 of 10 health districts: Limbe, Buea, and Tiko. The first cases originated from maritime health districts along the Nigeria-Cameroon border, and spread progressively in-country over time, with an exponential rise in number of cases in mainland health districts following pipe-borne water interruptions. Case fatality was higher in maritime health districts (3.39%) compared to mainland districts (1.5%, *p* < 0.01). We did not find an association between overcrowding and cholera, but the results suggest a potential dose-response relationship with an increasing number of household members (>5 people: (crude OR 1.73, 95% CI 0.97–3.12) and 3–5 people: (crude OR 1.47, 95% CI 0.85–2.60)), even after adjusting for internally displaced status and number of household compartments in the multivariable model (aOR 1.54, 95% CI 0.80–3.02).

**Conclusions:**

We report the largest cholera outbreak in the Southwest region. Our findings suggest the cross-border spread of cases from the Nigerian outbreak, likely driven by overcrowding in major cities. Our study highlights the need for cross-border surveillance, especially during humanitarian crises.

## Background

Cholera is a highly contagious bacterial disease transmitted through contaminated food and water. It is a major public health problem worldwide, estimated by 2015 to affect over 1.3 to 4 million people each year [1]. The causative agent *Vibrio cholerae* is divided into more than 200 serogroups [2], with outbreaks typically caused by toxigenic strains of the serogroups O1 and O139 [3]. Populations at greatest risk are those with limited access to clean water and sanitation, while factors such as conflict, unplanned urbanisation, population displacement and climate change further increase the risk of outbreaks [4, 5].

After years of decline in cholera incidence, the 2021-22 period was marked by a global resurgence of the disease [6, 7]. Over 23 countries reported cholera epidemics in 2021 and 30 countries in 2022. Some countries, such as Nigeria, experienced their largest ever outbreaks, while others, such as Lebanon, experienced an outbreak for the first time in almost 20 years [6, 8, 9]. Sub-Saharan Africa especially faced an exponential rise in number of cases [10]. Several countries in the region reported concurrent epidemics often accompanied by cross-border spread [11]. The factors associated with the global rise in cases remain unexplained; however, conflict and global climate crises may have played a crucial role [10, 12, 13].

Cameroon has experienced recurring cholera outbreaks since 1991 with an increasing annual incidence [14]. The largest outbreak was experienced in 2011 (23,152 reported cases and 843 deaths). Although studies on seasonality are sparse, cholera transmission in Cameroon is predicted to rise in May and October [15], coinciding with the rainy season, which extends from May to November [16]. Over the years, most cases have been notified by northern parts of the country with a high case-fatality rate of up to 8%.[17–19] The northern regions are marked by long-standing humanitarian crises, semi-arid climate, difficulties maintaining adequate hygiene and limited access to healthcare [17, 20, 21]. In the southern parts of the country, the Littoral region, particularly Douala, the economic capital, has also been a hotspot for cholera. This is due to brackish water reservoirs, poor quality of water, and sanitation compounded by a high population density [22]. The Southwest region has recorded an estimated 8% of the total number of cases over the years [19].

In October 2021, a new outbreak of cholera was declared in Cameroon that disproportionately affected the Southwest region. By March 2022, 6652 cases and 134 deaths had been notified nationwide [9], of which over 4400 cases (68%) were living in the Southwest region. The majority of cases in the Southwest region were reported in the cities of Limbe, Buea, and Tiko. Concurrently, socio-political instability in the region had led to an influx of hundreds of thousands of internally displaced persons (IDPs), with Limbe, Buea, Tiko, and Kumba accommodating the highest numbers in the region [23, 24]. In addition to the high influx in these cities, regular trans-border movements and trade, as well as cross-cultural communal living that occurs along the Cameroonian/Nigerian border, facilitated thousands of Cameroonians to flee to Nigeria during the crisis [24]. Meanwhile, Nigeria was also facing an unprecedented outbreak of cholera during the same period with over 112,000 cases and 3,635 deaths (CFR 3.2%) by March 2022 [9].

Here, we conducted an epidemiological investigation of the spread of cholera and associated factors in the Southwest region of Cameroon during the 2021-22 outbreak to illustrate the importance of cross-border investigation and control for a more efficient response to cholera outbreaks.

## Methods

### Study design

We described the outbreak in time, place and person based on a retrospective analysis of surveillance data collected in the form of a line list, between October 2021 and July 2022 when the last outbreak case occurred in the region. Furthermore, we carried out a case-control study to explore the drivers associated with the outbreak in the most affected cities.

### Setting

**Fig. 1:**
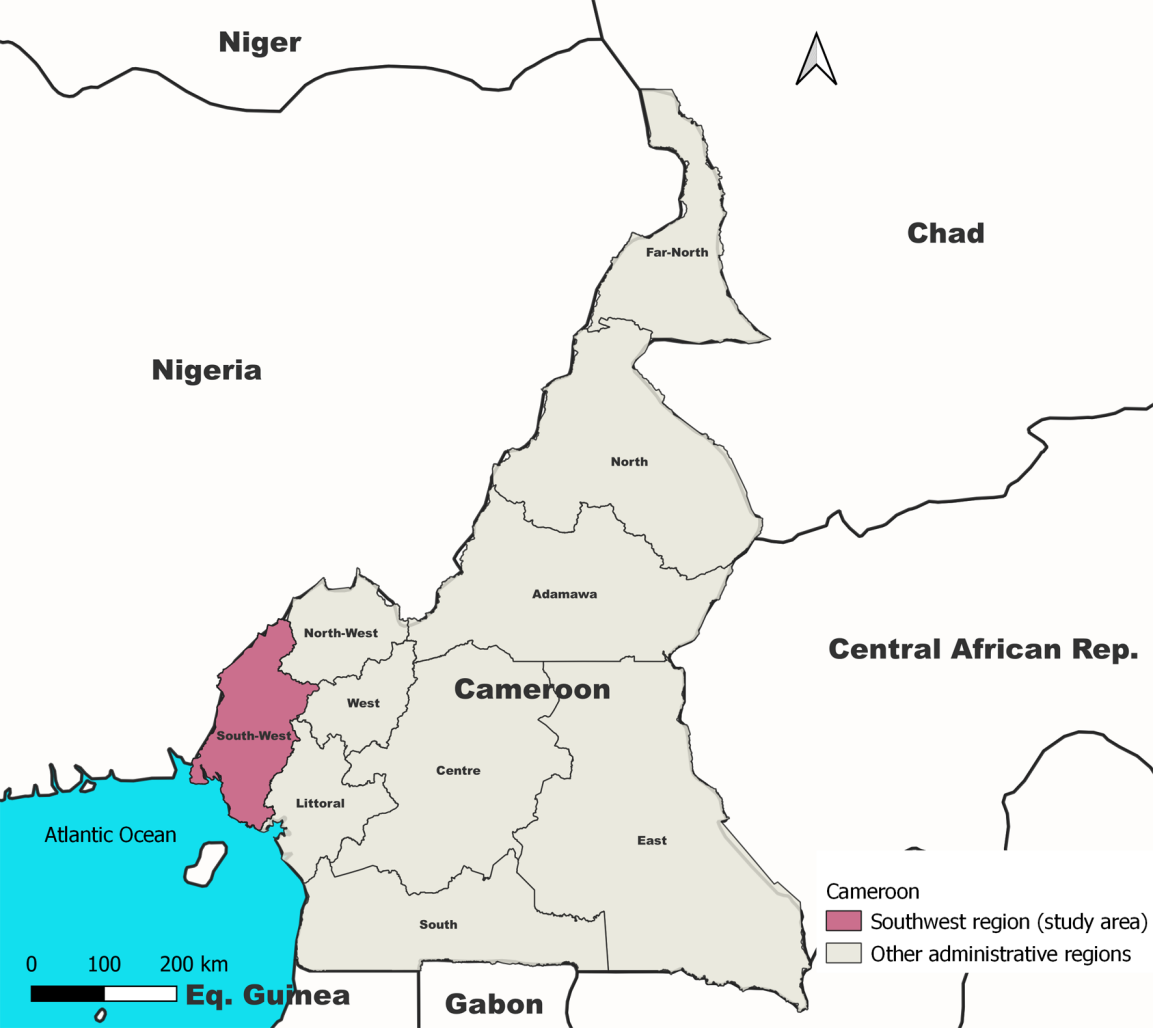
Map of Cameroon showing the Southwest Region and other administrative boundaries

The Southwest region is one of the 10 administrative regions of Cameroon. It is located on the west coast of the country sharing boundaries with the Cross River State in Nigeria and the Atlantic Ocean (Fig. 1).

The Southwest region is further divided into two main health intervention zones: the maritime zone and the mainland zone. The maritime zone, including the health districts in and around the Bakassi peninsula, is made up of predominantly rural health districts and has several extensions of the ocean inland. These communities are closest to the Nigerian border. The mainland zone includes health districts such as Limbe, Buea, Tiko, Muyuka, and Kumba, farther from the Bakassi peninsula and also share a border with the Atlantic Ocean, but no major extensions of the sea inland. In contrast to maritime health districts, mainland districts are predominantly urban.

Fishing is widely practiced by both Nigerian and Cameroonian residents in communities in the maritime district, with a high catch rate of up to 70 kg of fish per boat per day recorded during peak months in the Bakassi peninsula alone [25]. Despite the historically protracted war between Cameroon and Nigeria over sovereignty of the oil-rich Bakassi peninsula border area, this was never an impediment to frequent cross-border movement and trade [26].

A humanitarian crisis has been ongoing in the Southwest and Northwest regions of Cameroon since 2016 [27, 28]. Residents have fled from highly remote communities and settled in large urban cities such as Limbe, Buea, and Tiko in the Southwest, and also in the Littoral and other regions. Some equally fled to neighboring Nigerian states [28]. Limbe, Buea, and Tiko host more than a hundred thousand internally displaced persons due to the aforementioned crises [23]. Limbe and Buea faced massive overcrowding due to the crisis. In Buea, the population growth rate rose to 42% in 2020 compared to 5.6% in 2005 [23].

### Cholera surveillance in Cameroon

Cholera is a reportable disease in Cameroon. The threshold to declare an epidemic is one laboratory confirmed case via culture or PCR [29]. Following the confirmation of a case, the cholera incident management system is activated, surveillance teams in communities and healthcare facilities are trained in case identification, data collection and other response activities. Paper-based data is collected at the community level and health facilities, collated in Microsoft Excel at the health district level, and forwarded to the regional delegation of health, from where the data is directed to the central level at the Ministry of Health, instigating appropriate targeted outbreak response.

### Outbreak description from routine epidemiological data

To describe this outbreak, we gathered pseudonymized surveillance data from the Southwest Regional Delegation of Public Health. At the start of the outbreak, cases were notified and registered based on the following case definitions:

*Suspected case:*

Anyone presenting with diarrhoea with/without vomiting in Ekondo Titi, Bakassi, Mbonge, Limbe, Buea, Kumba, and Muyuka Health Districts from the date of confirmation of the first case in the health district.

*Confirmed cases:*

Any suspected case from whom Vibrio cholerae O1 or O139 has been isolated in stool by culture or PCR.

This case definition was gradually contextualized according to the evolution of the outbreak, by including newly affected health districts. Data were presented in the form of a line list of all reported suspected and confirmed cholera cases between October 2021 and July 2022, when the last case was notified in the region. An ongoing outbreak in other regions of the country kept the Southwest region in alert mode and regional health authorities therefore did not declare an end of the outbreak.

Registered in the line list were demographic characteristics, profession, affected health district, health area, cholera diagnosis, date of onset, clinical management, and patient outcomes. For laboratory confirmation, liquid stool samples or rectal swabs were collected and transported in Cary-Blair medium to the laboratory of the Laquintinie Hospital in Douala for microbiological culture. The isolates were characterised and strain identification for Inaba or Ogawa was confirmed using polyvalent antisera for *Vibrio cholerae* O1 and O139. Due to logistical challenges, only a subset of samples of suspected cases of cholera was sent for confirmatory testing.

Throughout the rest of the manuscript, we define cholera cases as the combination of suspected and confirmed cholera cases.

### Case-control study to identify associated risk factors

To identify factors associated with suspected cholera in the most affected health districts, we set up a case-control study to compare the characteristics of reported cholera suspected or confirmed cases with those of neighbourhood controls. Interviewing of cases and controls was conducted between 26 January and 4 February 2023 in Limbe, Buea, and Tiko, five months after the outbreak had ended in the region.

To determine a suitable sample size for the case-control study, we used Dupont’s formula [30], which requires information on power (probability of detecting a real effect), on the alpha value (probability of detecting a false effect), on the correlation coefficient (ϕ) for exposure between matched cases and controls, on the probability of exposure in the control group, on the number of control subjects matched to each case subject and finally on the expected odds ratio (ψ).

We hypothesised that internally displaced persons and/or people living in overcrowded conditions may be at greater risk of cholera, and we intended to detect an odds ratio of 2 with 80% power and an alpha of 0.05. We assumed a probability of exposure among controls of 30% and set the correlation coefficient ϕ for exposure between matched cases and controls at 0.2 in the absence of prior data [30]. With these assumptions, we arrived at a sample size of 348 participants, 174 cases and 174 matched controls.

Although we planned to conduct a one-to-one matched case-control study, on the basis of age, sex, and residence, when collecting the data, it was clear that this matching was not feasible in the field and data was collected for a regular case-control study. Note that for an unmatched case-control study, the desired sample size with the same assumptions and requirements, calculated with the Fleiss method with continuity correction, would have been 306 participants (153 in each group). The rest of this manuscript presents an unmatched case-control study.

For inclusion, recovered suspected cases of cholera were randomly approached by community health workers responsible for cholera surveillance in the locality by going to their home. For each case, an individual was selected from a neighbouring household who did not previously have self-reported cholera during the outbreak.

Cases and controls were administered an identical validated questionnaire, developed by the UNICEF Integrated Analytics Cell (CAI – formerly CASS) and partners, initially designed to understand cholera transmission in the Democratic Republic of Congo, and adapted for Cameroon [31]. The questionnaire included information on demographics, residency, migration history, accommodation, and known risk factors such as unsafe drinking water, poor sanitation, crowding, and health literacy.

## Data collection

Data for the outbreak description were compiled by the health authorities through the surveillance system described above. Data for the case-control study were collected electronically using KoboCollect (https://www.kobotoolbox.org).

### Definition of variables

We defined type of house/residence under 3 categories as follows: one compartment (a single room, including living and sleeping space, plus or minus bathroom), a two compartment household (a separate living room and single bedroom plus or minus bathroom), three or more compartments (separate living room and 2 or more bedrooms +/- bathroom). We categorised the number of household members into three categories: <3, 3–5, and >3. We classified individuals who had relocated over the last 5 years into 5 categories: those who relocated and lived in a group accommodation, relocated and living in home of a relative, relocated and self-settled, or other (one who relocated but does not fall in the above categories, and stable accommodation for individuals who did not relocate over the last 5 years.

### Data analysis

For the description of the outbreak, all suspected and confirmed cases of cholera registered in the line list were considered for descriptive analyses. Attack rates in maritime and mainland communities were calculated as the cumulative total number of cases per type of community, divided by the total population at risk (2021 population estimate) expressed as a percentage. Fisher exact test was used to test the difference in the mortality between maritime and mainland communities affected by cholera.

In the case-control study, we conducted unmatched logistic regression using the generalised linear model. We calculated odds ratios (OR), their 95% confidence interval (95% CI) for models including individual exposures, and adjusted odds ratios (aOR) and 95% CI for models including multiple exposures. Exposures included overcrowding (measured by the number of people living in the same household, in categories of less than 3, 3 to 5 and more than 5 people per household), profession/trade, internally displacement, number of compartments in a house and drinking water sources. We were particularly interested in assessing the existence of an association between overcrowding and cholera. For this, we built a directed acyclic graph (DAG) to identify confounders on the causal pathway. On the basis of the DAG, we determined which variables to control for in the multivariable analysis.

Data cleaning and analysis of both datasets were performed using R statistical software [32]. Maps were generated using QGIS [33], and R. The shapefiles for the generation of map in QGIS were available from the Humanitarian Data Exchange service provided by the Office for the Coordination of Humanitarian Affairs (OCHA) at https://data.humdata.org/, under a CC-BY 4.0 licence.

## Results

### Spread of cholera during the 2021-22 cholera outbreak

#### Description of the affected population

Between October 15, 2021 and July 21, 2022, a total of 6023 (55.6%) cases were recorded, of which 3268 (54%) were men, and 93 deaths (case fatality 1.5%) were recorded in the Southwest region. Stool cultures were positive for *Vibrio cholerae* O1, Inaba serotype, in 24 out of 30 patient samples. The overall attack rate in the region was 0.5%. The median age of the affected persons was 27 years (IQR 18–40). Four hundred and forty (7.2%) were children under 5 years old, while 823 (13.6%) were over 50 years old. While 5344 (89%) cases were reported from mainland health districts (attack rate 0.5%), 679 (11%) were notified in maritime health districts (attack rate 0.3%). Demographic and clinical characteristics are summarised in Table 1.

**Table 1:** Demographic and clinical characteristics of suspected cases of cholera during the 2021-22 in the Southwest Region

Characteristic	*N* = 6023^*1*^
**Sex**	
Female	2755 (46%)
Male	3268 (54%)
**Age**	
Median	27 (18, 40)
Missing	22
**Outcome**	
Alive	5930 (98.5%)
Dead	93 (1.5%)
**Hospitalized**	
Yes	5061 (84%)
No	958 (15.9%)
Dead on arrival	4 (< 0.1%)
**Duration of Hospitalization**	
Median (in days)	2 (1,3)
**Dehydration severity**	
Mild	1152 (19%)
Moderate	3149 (52%)
Severe	1717 (29%)
Missing	5 (< 0.1)
**Vomiting**	
Yes	4380 (72.7%)
No	1315 (21.8%)
Missing	328 (5.5%)
**Diarrhea**	
Yes	5989 (99.4%)
No	28 (0.5%)
Missing	6 (< 0.1%)
**Health district affected**	
**Maritime health districts**	
Ekondo Titi	348 (5.8%)
Bakassi	309 (5.1%)
Mundemba	18 (0.3%)
Mbonge	4 (< 0.1%)
**Mainland health districts**	
Limbe	3127 (52%)
Buea	942 (16%)
Tiko	906 (15%)
Kumba South	215 (3.6%)
Muyuka	145 (2.4%)
Kumba North	6 (0.1%)
Mamfe	3 (< 0.1%)

^*1*^ Median (IQR); n (%)

**Fig. 2:**
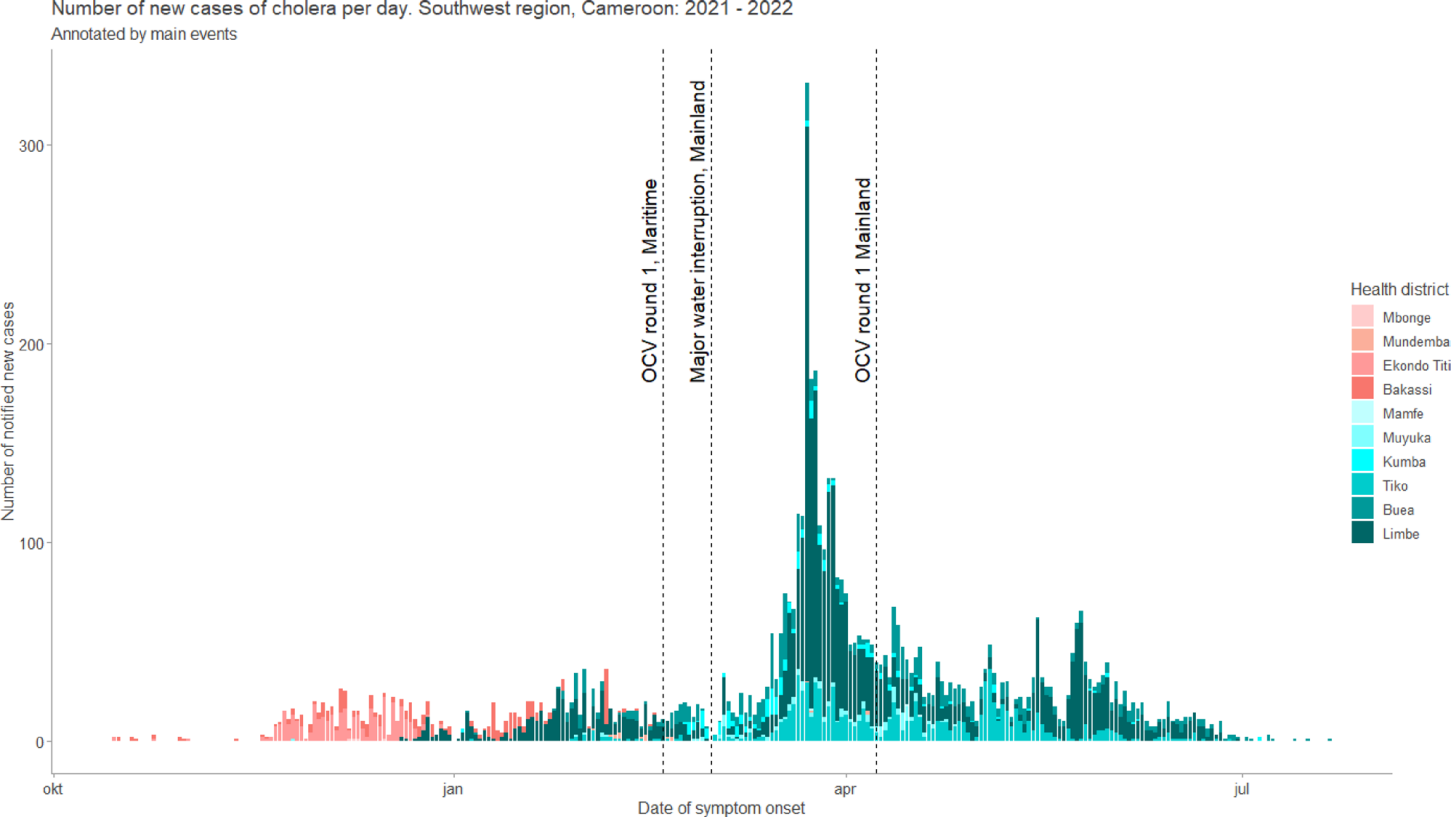
Epidemiological curve of daily reported cholera cases per notifying health district, Southwest region, Cameroon. *The red theme colours show the maritime outbreak while the green theme colours show the mainland outbreak*

Eleven of eighteen health districts in the region reported at least one case of cholera. Most cases (4975/6023, 82.6%) were notified by 3 of 11 health districts, Limbe, Buea and Tiko, which are all urban mainland districts (Fig. 2).

**Fig. 3:**
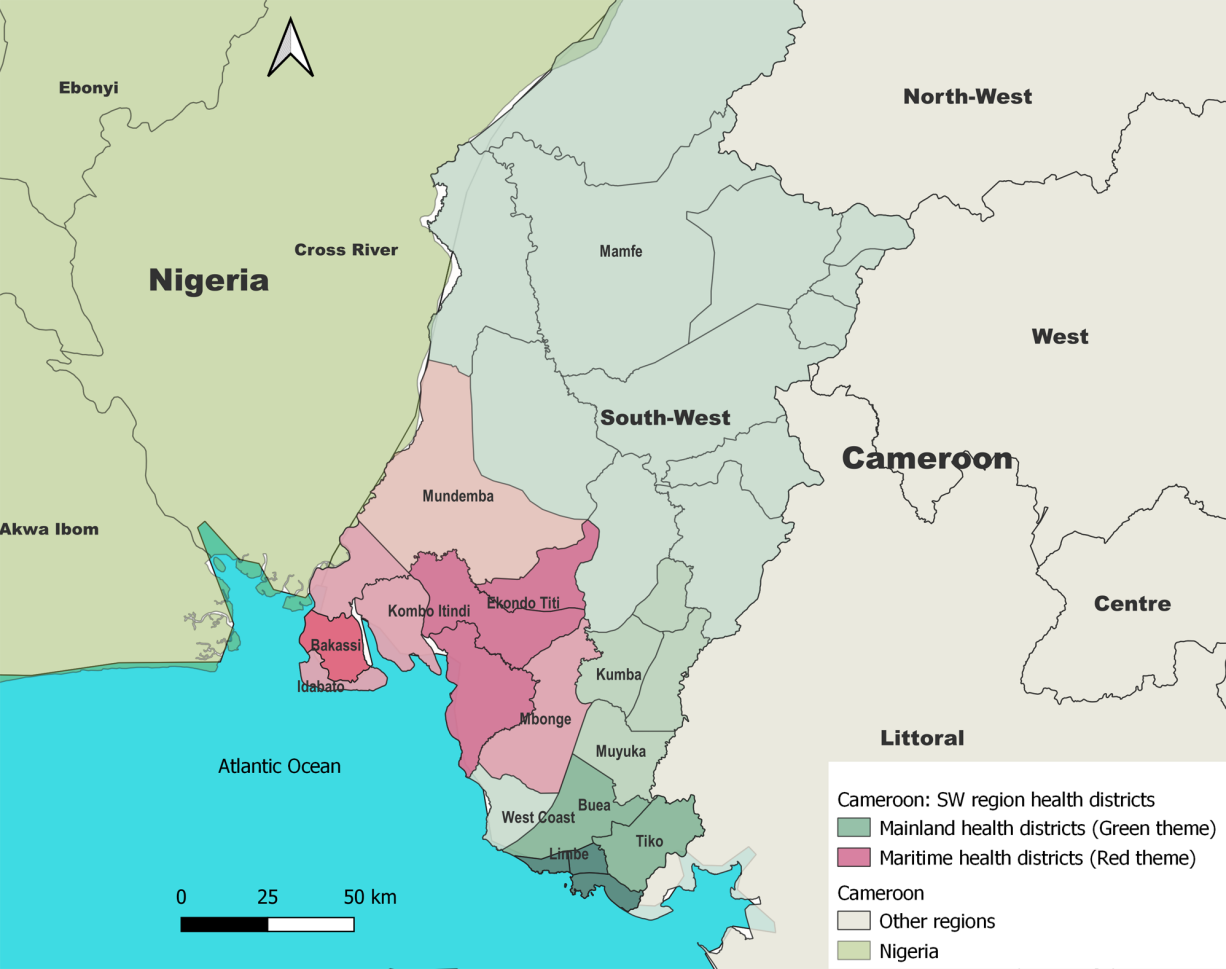
Spatial distribution of notified cholera cases in the Southwest region of Cameroon, 2021–2022. The red theme colours show the maritime outbreak, while the green theme shows the mainland outbreak. The darker the colour, the more intense the incidence. Only districts with at least one case are named on the map

Ten of the first 20 cases notified during the first month of the outbreak were fishermen. The spatial distribution of the notified cholera cases in the Southwest region is shown in Fig. 3.

### Origin of first cases and progression of the outbreak over time

The first cases originated from maritime communities along the Nigeria-Cameroon border (Oron and Andoni, Nigeria; Kesse, Cameroon). This started a predominantly maritime outbreak with a low but sustained incidence with a maximum of 26 cases per day by the end of December 2021. Although 4 maritime health districts were affected, 657 of 679 (96.8%) of the maritime outbreak cases were reported by two health districts Bakassi and Ekondo Titi health districts.

On 21 December 2021, a first urban case was notified in the Limbe health district, marking the start of a predominantly mainland outbreak. By the end of February 2022, the number of cases in mainland districts had risen to 735 while the number of cases in the maritime districts declined. Later, a major interruption of potable water supply to mainland was followed by an exponential increase in number of cases, with the highest number of cases in a single day (331) reported on 23 March 2023. Almost all cases (330 of 331) were from mainland health districts.

### Case fatality rate (CFR)

The overall CFR was 1.5% (93/6023). The majority (57/93, 61.3%) of notified deaths were male. The deceased were predominantly older, median age 40 years (IQR 25–57); 43 years in females and 37 in males. The case fatality was significantly higher in the maritime health districts (3.4%) affected towards the start of the outbreak compared to the mainland districts (1.5%, *p* < 0.01). Nine out of the 93 total deaths were from children under 5 years of age. Seven out of the nine child deaths were children from maritime health districts.

### Clinical characteristics, case management, and outbreak response

During the outbreak, 5061 (84%) cases were hospitalised. While 1717 (29%) of the cases were severe, 3149 (52%) moderate, and 1152 (19%) were mild. The median delay between symptom onset and hospitalization was less than 24 h (IQR 0–1 day), while the median duration of hospitalisation was 2 days (IQR 1–3 days). The patients were treated with oral rehydration solution, antibiotics, and administration of intravenous fluid or zinc, or a combination. Eighty-one out of 93 deaths or 87.1% were notified as severe cases and 4 patients died upon arrival (Table 1).

Response activities included training of community health workers on case identification, community case management of mild cases, water, sanitation, and hygiene (WaSH) intervention which included the use of aquatabs, disinfection of households of cases and neighbours, hand washing, in addition to community sensitisation and mobilization.

Two rounds corresponding to two doses of oral cholera vaccine (OCV) were administered in a case area targeted intervention (CATI) vaccination system. The first round of OCV administration was conducted between 18 and 22 February 2022 in maritime communities and 8 to 12 April 2022 in mainland communities. The second round of OCV was conducted between 31 August and 4 September 2022 for maritime districts and 8 to 12 October 2022 for mainland districts. The proportion of people vaccinated compared to the target population was ~ 94% in maritime communities and ~ 98% in mainland communities.

### Risk factors and community dynamics

#### Demographic characteristics of the case-control study population

A total of 207 recovered cholera cases (129, 62% female) and 206 (127, 60% female) controls were administered questionnaires. 63% (260 out of 413) were recruited from the Limbe health district, 14% (58 out of 413) from Buea and 23% (95 out of 413) from the Tiko health district. 89% of the cases and 91% of the controls were between 18 and 49 years of age. 25% (51 out of the 207) of cases and 28% (57 out of the 206) of controls were internally displaced due to conflict. More than 35% of the cases and controls had no monthly income. Among the cases, 59% lived in a one- or a two compartment household versus 69% of the controls; meanwhile 39% of the cases and 33% of controls lived with more than 5 members in the household. In some households, up to 17 people lived together in a single compartment house. (Table 2).

**Table 2:** Summary of household size per type of house/residence

Type of house/residence	Summary of household size (based on the number of household members)			
	Mean	Median	Min	Max
Three or more compartments (separate living room and two or more bedrooms +/- bathroom)	6.24	6	1	15
Two compartments (separate living room and a single bedroom +/- bathroom)	4.26	4	1	10
One compartment (single room including bedroom and living space +/- bathroom)	4.13	4	1	17

The demographic characteristics of the cases and controls are summarised in Table 3.

**Table 3:** Demographic characteristics of cases and controls

Characteristic	Case, *N* = 207^*1*^	Control, *N* = 206^*1*^
**Age group**		
18–49 years	185 / 207 (89%)	188 / 206 (91%)
>= 50 years	22 / 207 (11%)	18 / 206 (8.7%)
**Sex**		
Female	129 / 207 (62%)	127 / 206 (60%)
Male	78 / 207 (38%)	79 / 206 (40%)
**Health district**		
Buea	32 / 207 (15%)	26 / 206 (13%)
Limbe	127 / 207 (61%)	133 / 206 (65%)
Tiko	48 / 207 (23%)	47 / 206 (23%)
**Internally displaced due to conflict**		
No	156 / 207 (75%)	149 / 206 (72%)
Yes	51 / 207 (25%)	57 / 206 (28%)
**Number of household members living together**		
<3	28 / 206 (14%)	40 / 201 (20%)
3–5	98 / 206 (48%)	95 / 201 (47%)
>5	80 / 206 (39%)	66 / 201 (33%)
Missing	1	5

^*1*^ n / N (%)

In a univariable analysis, we could not find an association between overcrowding, defined as the number of people within the household and cholera, while point estimates of the crude OR were higher in households with more than 5 people (odds ratio (OR) 1.73, 95% confidence interval (CI) 0.97–3.12) and (OR 1.47, CI 0.85–2.60) in households of 3 to 5 people, compared to those with less than 3 people. We were equally unable to detect higher odds in internally displaced persons (OR 0.85, 95% CI 0.55–1.33). The following factors were protective in a univariable analysis: living in a two compartment household compared to an apartment with three or more compartments (OR 0.49, CI 0.29–0.82), the consumption of borehole (OR 0.46, CI 0.21–0.95) and well water (OR 0.31, CI 0.13–0.74) during periods of water scarcity compared to tap water. (Table 4)

**Table 4:** Factors associated with reported cholera

Characteristic	case, *N* = 207^*1*^	control, *N* = 206^*1*^	OR^2^	95% CI^3^	aOR^4^	95% CI^3^
**Age**						
18–49 years	185 / 207 (79%)	188 / 206 (91%)	—	—		
>= 50 years	22 / 207 (11%)	18 / 206 (8.7%)	1.24	0.65,2.42		
**Sex**						
Female	129 / 207 (62%)	127 / 206 (62%)	—	—		
Male	78 / 207 (38%)	79 / 206 (38%)	0.97	0.65, 1.45		
**Occupation**						
Employed but not specified	104 / 207 (50%)	108 / 206 (52%)	—	—		
Agriculture/forestry	20 / 207 (9.7%)	18 / 206 (8.7%)	1.15	0.58, 2.32		
Fishing	2 / 207 (1.0%)	2 / 206 (1.0%)	1.04	0.12, 8.78		
Healthcare	2 / 207 (1.0%)	6 / 206 (2.9%)	0.35	0.05, 1.54		
Retail	1 / 207 (0.5%)	2 / 206 (1.0%)	0.52	0.02, 5.50		
Student	0 / 207 (0%)	1 / 206 (0.5%)	0.00	0.00, 0.00		
Trade	9 / 207 (4.3%)	3 / 206 (1.5%)	3.12	0.90, 14.3		
Unemployed	69 / 207 (33%)	66 / 206 (32%)	1.09	0.70, 1.67		
**Internally displaced due to conflict**						
No	156 / 207 (75%)	149 / 206 (72%)	—	—	—	—
Yes	51 / 207 (25%)	57 / 206 (28%)	0.85	0.55, 1.33	0.86	0.53, 1.38
**Relocation in the last 5 years and type of settlement**						
Stable accommodation (did not relocate during the past 5 years)	114 / 202 (56%)	120 / 196 (61%)	—	—		
Relocated and living in Group accommodation	3 / 202 (1.5%)	1 / 196 (0.5%)	3.16	0.40, 64.3		
Relocated and living in home of relative/other	31 / 202 (15%)	24 / 196 (12%)	1.36	0.75, 2.47		
Relocated and self-settled	51 / 202 (25%)	48 / 196 (24%)	1.12	0.70, 1.79		
Other	3 / 202 (1.5%)	3 / 196 (1.5%)	1.05	0.19, 5.79		
Missing	5	10				
**Type of house/residence**						
Three or more compartments (separate living room and two or more bedrooms +/- bathroom)	86 / 207 (42%)	64 / 206 (31%)	—	—	—	—
Two compartments (separate living room and a single bedroom +/- bathroom)	39 / 207 (19%)	59 / 206 (29%)	0.49	0.29, 0.82	0.63	0.35, 1.13
One compartment (single room including bedroom and living space +/- bathroom)	82 / 207 (40%)	82 / 206 (40%)	0.74	0.48, 1.16	0.86	0.51, 1.46
Missing	0	1				
**Number of household members**						
<3	28 / 206 (14%)	40 / 201 (20%)	—	—	—	—
3–5	98 / 206 (48%)	95 / 201 (47%)	1.47	0.85, 2.60	1.41	0.78, 2.59
>5	80 / 206 (39%)	66 / 201 (33%)	1.73	0.97, 3.12	1.54	0.80, 3.02
Missing	1	5				
**Main drinking water source**						
Tap water (Public)	97 / 207 (47%)	94 / 204 (46%)	—	—		
Borehole	34 / 207 (16%)	35 / 204 (17%)	0.94	0.54, 1.63		
Mineral water	7 / 207 (3.4%)	1 / 204 (0.5%)	6.78	1.18, 128		
Protected Well	3 / 207 (1.4%)	4 / 204 (2.0%)	0.73	0.14, 3.38		
Surface water	18 / 207 (8.7%)	12 / 204 (5.9%)	1.45	0.67, 3.26		
Tap water (Within Household)	45 / 207 (22%)	55 / 204 (27%)	0.79	0.49, 1.29		
Unprotected Well	3 / 207 (1.4%)	3 / 204 (1.5%)	0.97	0.18, 5.35		
**Water available at the time of interview**						
Yes	162 / 206 (79%)	171 / 205 (83%)	—	—		
No	44 / 206 (21%)	34 / 205 (17%)	1.37	0.83, 2.26		
Missing	1	1				
**Type of water storage vessel**						
Jerrycans/gallons	103 / 179 (58%)	108 / 191 (57%)	—	—		
Bucket (distributed during the sanitary cordon)	24 / 179 (13%)	22 / 191 (12%)	1.06	0.54, 2.09		
Bucket (other origin)	42 / 179 (23%)	50 / 191 (26%)	0.77	0.45, 1.29		
Other	10 / 179 (5.6%)	11 / 191 (5.8%)	1.12	0.43, 2.94		
Missing	55	39				
**Water source during water scarcity episodes**						
Tap water	24 / 207 (12%)	13 / 206 (6.3%)	—	—		
Borehole	72 / 207 (35%)	85 / 206 (41%)	0.46	0.21, 0.95		
Bottled water	12 / 207 (5.8%)	13 / 206 (6.3%)	0.50	0.17, 1.40		
No water	4 / 207 (1.9%)	3 / 206 (1.5%)	0.72	0.14, 4.13		
Spring	44 / 207 (21%)	31 / 206 (15%)	0.77	0.33, 1.72		
Unreported	32 / 207 (15%)	28 / 206 (14%)	0.62	0.26, 1.43		
Well water	19 / 207 (9.2%)	33 / 206 (16%)	0.31	0.13, 0.74		
**Travelled to Nigeria in the last one year**						
No	62 / 63 (98%)	54 / 58 (93%)	—	—		
Yes	1 / 63 (1.6%)	4 / 58 (6.9%)	0.22	0.01, 1.53		
Missing	144	148				

^*1*^ n / N (%), ^*2*^ OR = Odds Ratio, ^*3*^ CI = Confidence Interval, ^*4*^ OR adjusted for internally displaced status and type of housing/residence

**Fig. 4:**
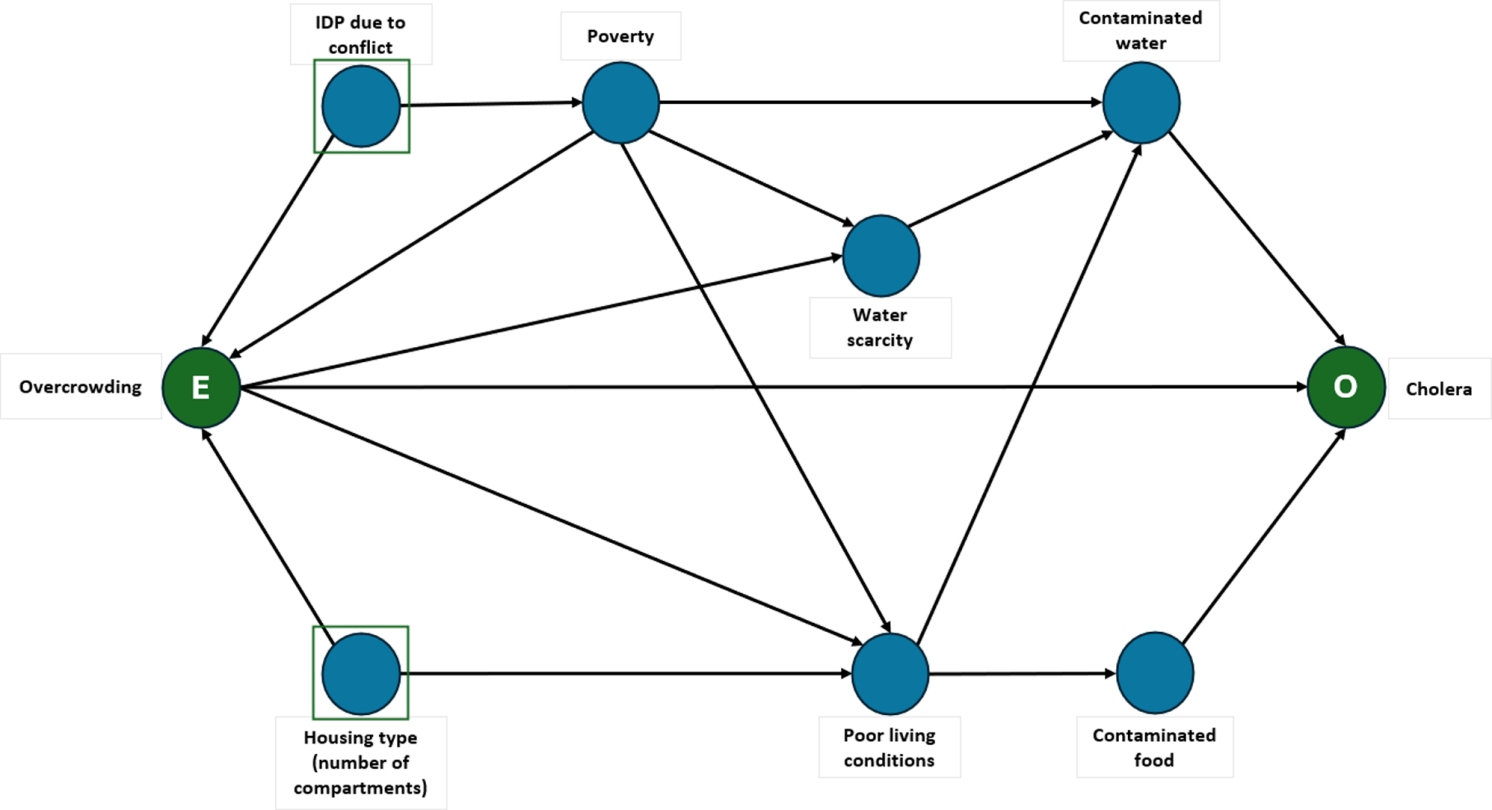
Directed acyclic graph to guide the selection of variables along the causal pathway. Green circles represent the exposure and outcome of interest. E. Exposure: Overcrowding defined by the number of household members O. Outcome: Cholera Blue circles represent confounding variables on this causal pathway. Squares (around blue circles) represent the minimally sufficient set that was adjusted for in the multivariable model

In a multivariable analysis, we equally found no association between overcrowding and cholera after controlling for internally displaced status and type of housing/residence (number of household compartments). Figure 4.

We obtained adjusted odds ratio (aOR) for overcrowding: 1.54 (95% CI 0.80–3.02) for living with more than 5 people in a household and aOR 1.41 (95% CI 0.78–2.59), for living with 3 to 5 people in a household compared to living with less than 3 people.

## Discussion

Our study describes the largest outbreak of cholera ever recorded in the Southwest region of Cameroon. Within 1 year, more than 6000 cases were notified, out of a total of ~ 11,000 cases throughout the country. The first cases were notified along the Cameroonian-Nigerian border in predominantly maritime communities and spread in-land towards mainland health districts, followed by an unprecedented mainland outbreak. Our case-control investigation points toward overcrowding as a potential driver behind the high number of cholera cases in mainland cities.

Meanwhile, across the border, Nigeria was equally facing an unprecedented outbreak of cholera which had begun since 2020 [4]. At the time that coincided with the start of the outbreak in Cameroon, the epicentre of the Nigerian outbreak was the Cross River state of Nigeria, which shares boundaries with the Southwest region of Cameroon and also harbors tens of thousands of Cameroonian refugees [24]. Additionally, significant transborder movements and trade occur along this border, particularly among fishing communities of both countries. The initial cases notified in Cameroon originated from Oron and Andoni (Cross River state, Nigeria) and from Kesse (Maritime health district, Cameroon), and consequent progressive spread of cases towards further communities supports the hypothesis of potential cross-border transmission. Despite very frequent transborder movements in the southern parts of Cameroon, this is, to the best of our knowledge, the first reported outbreak likely due to cross-border spread in this region.

The burden on the Southwest region was unprecedented. With over 50% of the total number of nationally reported cases, the disproportionate impact on this region was striking. Although the region has experienced cholera outbreaks in the past, its cumulative contribution to the national case load never exceeded 8% [19]. This outbreak therefore starkly contrasts with historical patterns, where the three regions—Far North, North, and Littoral—typically accounted for a cumulative 80% of the total number of cholera cases reported in the country [19].

The proportion of reported cases was slightly higher in males compared to females (54% versus 46%). The majority of deaths equally occurred in males (61.3%) and primarily in older adults, with a median age of 40. This differs from endemic settings, where most cases occur in young children, possibly due to immunity in adults. Although the overall case fatality (1.54%), in the Southwest was generally lower than during previous outbreaks of cholera in Cameroon [34], it however stayed above the recommendation of the World Health Organisation for a well managed cholera outbreak (<1%) [35]. The maritime communities which experienced a significantly higher case fatality (3.4%), and were affected during the start of the outbreak, were possibly unprepared for an outbreak of cholera. Additional logistical challenges might have made it difficult for health authorities to mount a rapid outbreak response in that area. However, an overall decline in mortality over time was consistent with other findings in which mortality rates from cholera were shown to decline over the course of the epidemics [17, 18, 34].

Meanwhile, this outbreak coincided with a period of socio-political crisis in the Southwest region, that led to an influx of conflict-induced internally displaced persons settling with relatives or sought their own accommodation in major cities. In some instances, up to 17 individuals lived in single compartment households in our study. Although this influx resulted in near-empty villages in some especially rural areas, it led to higher population densities in surrounding urban cities that face less insecurity, with up to 42% growth rate observed in cities such as Buea in 2020 [23].

In our case-control study, we did not observe a relationship between overcrowding and cholera. The increase in point estimates, albeit having wide confidence intervals, might point towards a weaker association than the OR of 2 for which our study was powered to detect. Therefore, overcrowding may have played a role in driving the epidemic in mainland cities, consistent with other findings [23, 36]. Similarly, we could not find an association between cholera and being internally displaced due to conflict as in other settings [37]. This was likely due to the fact that IDPs in the Southwest region lived in similar conditions as the residents, rather than in camps or other specific communal lodging [23, 37].

Besides overcrowding, the first weeks of 2022 was marked by an unprecedented scarcity of potable water in Limbe, Buea, and Tiko, already reported by Monono and colleagues as well as other media outlets [23, 38]. Several inhabitants resorted to alternative sources of drinking water during the period. Our study found that borehole and well water were protective for cholera compared to tap water. Several wells and boreholes were rehabilitated, constructed and/or decontaminated as part of the WaSH outbreak response. Furthermore, aquatabs were distributed in affected communities for water treatment prior to consumption. As a less reliable water source, well and borehole water are likely to be treated before consumption, compared to tap water, which is considered a more reliable potable water source in these communities.

### Limitations

Our study had a couple of limitations. First, we intended to set up a matched case-control study, but later went for an unmatched design. Second, we encountered delays in setting up the case-control study with participants recruited several months after the outbreak was over. This might have exposed our study to some recall or other sort of bias. Finally, contrary to the distribution of outbreak cases from the line list, where the majority (54%) were men, the majority of cases and controls recruited for the case-control investigation (64% for cases and 61% for controls) were women. However, we do not expect that this would have an effect on the inference we are making from our findings, as we do not expect big differences in housing conditions between men and women.

## Conclusion

In contrast to global control efforts towards the 2030 cholera roadmap, cholera cases have surged in several countries over the past few years with substantial cross-border spread. We conclude that this was potentially a cross-border outbreak, later driven by overcrowding in the most affected cities. Our study highlights the need for cross-border surveillance and enhanced outbreak preparedness especially in conflict-affected settings.

## Data Availability

Deidentified participant data collected or analyzed for this study will be made available by the corresponding author upon reasonable request.
